# Impact of Renal Replacement Therapy on Outcomes of Living Donor Liver Transplantation for Acute Liver Failure: A Cohort Study

**DOI:** 10.1155/2024/8422308

**Published:** 2024-09-05

**Authors:** Abu Bakar Hafeez Bhatti, Nauman ul Haq, Nayyer Mehmood, Danyal Hassan, Arsalan Ahmed, Wasim Tariq Malik, Haseeb Haider Zia, Mohammad Salih, Nusrat Yar Khan, Abid Ilyas, Nasir Ayub Khan

**Affiliations:** ^1^ Department of HPB Surgery and Liver Transplantation Shifa International Hospital, Islamabad, Pakistan; ^2^ Department of Nephrology Shifa International Hospital, Islamabad, Pakistan; ^3^ Department of Neurology Shifa International Hospital, Islamabad, Pakistan; ^4^ Department of Gastroenterology and Hepatology Shifa International Hospital, Islamabad, Pakistan; ^5^ Department of Surgical Critical Care Shifa International Hospital, Islamabad, Pakistan; ^6^ Department of Anesthesiology Shifa International Hospital, Islamabad, Pakistan

## Abstract

Despite the promising role of renal replacement therapy (RRT) in acute liver failure (ALF), high-risk patients need liver transplantation and remain at risk for death due to cerebral complications. The objective of this study was to report outcomes of living donor liver transplantation (LDLT) for ALF with perioperative RRT. This was a single-center retrospective cohort study. Out of 1167 LDLTs, 24 patients had ALF and met the King's College criteria for transplantation. They were categorized into no-RRT (*n* = 13) and RRT (*n* = 11) groups. We looked at 1-year posttransplant survival in these patients. The median serum ammonia level at the time of transplant in the no-RRT and RRT groups was 259.5 mcg/dL (222.7–398) and 70.6 mcg/dL (58.1–92.6) (*p* = 0.005). In the RRT group, serum ammonia level < 100 mcg/dL was achieved in all patients. Seven (53.8%) patients in the no-RRT group and 11/11 (100%) in the RRT group were extubated and regained full consciousness after LDLT (*p* = 0.013). The 90-day mortality was 6/13 (46.1%) and 2/11 (18.1%) (*p* = 0.211). There was no brainstem herniation-related mortality in the RRT group, that is, 5/13 (38.4%) and 0/11 (0%) (*p* = 0.030). The 1-year posttransplant survival was also significantly higher in the RRT group (*p* = 0.031). The use of RRT lowers serum ammonia levels and might reduce posttransplant mortality due to brainstem herniation.

## 1. Introduction

Acute liver failure (ALF) is a severe condition characterized by liver dysfunction, coagulopathy, and hepatic encephalopathy (HE) in patients without prior liver disease [1]. Mortality rates without treatment can be as high as 30%–50% [2, 3]. While some patients can recover with medical management, liver transplantation (LT) is often necessary for those with high-risk factors such as a high model for end-stage liver disease (MELD) score or meeting the King's College criteria (KCC) for ALF [1, 4, 5]. HE and cerebral complications are major contributors to mortality in ALF, and hyperammonemia is considered a relative contraindication to LT [1, 5–8]. Hyperammonemia predisposes to cerebral edema in ALF, and serum ammonia levels between 150 and 200 mcg/dL have been associated with neurological complications [9]. The empirical use of hyperventilation, hypothermia, mannitol, and hypertonic saline to reduce intracranial pressure has shown mixed results in improving mortality [10]. Extracorporeal systems, like plasma exchange and albumin dialysis, can serve as a bridge to recovery or transplant, but their routine use in ALF is not well-established [1, 2]. Renal replacement therapy (RRT) improves 21-day transplant-free survival in ALF and is indicated in patients with acute kidney injury or serum ammonia levels > 150 mcg/dL. Nevertheless, some high-risk patients may still require LT. It remains unclear if RRT improves posttransplant survival in this group [11–13]. While transplant outcomes with living donor liver transplantation (LDLT) for ALF are not frequently reported, it is an option in regions where deceased donation is limited [1]. In light of emerging evidence supporting the role of RRT in ALF, a protocol was developed for LDLT with perioperative RRT. The objective of the current study was to determine 1-year survival in patients with ALF who underwent LDLT with and without perioperative RRT.

## 2. Materials and Methods

This was a single-center retrospective cohort study and included patients who underwent LDLT for ALF. Between 2012 and 2023, 24/1167 (2%) transplants were performed for ALF. We defined ALF according to the European Association for the Study of the Liver (EASL) consortium, and patients with acute-on-chronic liver failure were excluded [14, 15]. The study was performed in accordance with the Declaration of Helsinki and STROBE guidelines.

**Figure 1:**
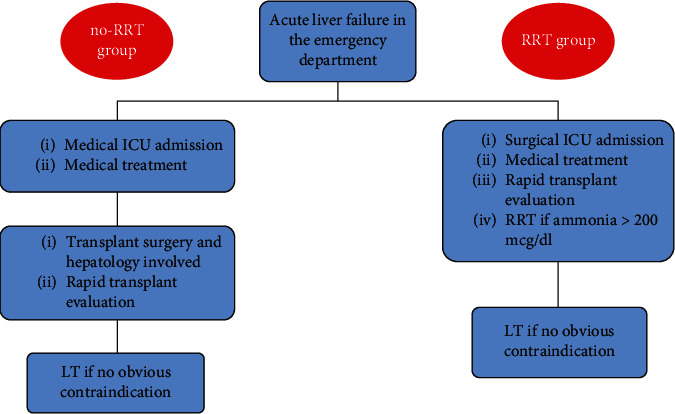
Changes in acute liver failure care pathway in the early (2012–2021) and later period (2021–2023).

Between 2012 and 2021, patients diagnosed with ALF were admitted to the medical intensive care unit (MICU). Standard ALF management was initiated, involving the transplant surgery and hepatology teams. Treatment for hyperammonemia was provided, and RRT was not used. Families were informed about the potential need for LT and asked to identify potential living donors. A LT was performed if the patient met the KCC for transplantation. However, delays in involving the transplant team were not uncommon, with some patients not being evaluated until 24–48 h after presentation (Figure 1).

### 2.1. ALF Pathway

Starting in March 2021, patients were screened in the emergency department, and if the family consented to a transplant, admitted to the surgical intensive care unit (SICU) under the care of the transplant team. Standard medical management was provided, a transplant workup was completed, and LT was performed if the patient met the KCC. Additionally, all patients with serum ammonia levels greater than 200 mcg/dL received RRT based on emerging evidence suggesting its benefits in improving transplant-free survival and correcting hyperammonemia [12, 13]. Typically, for RRT, a dialysate clearance of 35–40 mL/kg/h with a blood flow rate (Qb) of 200–250 mL/min was used. Ammonia levels were monitored regularly, and RRT intensity was adjusted if ammonia levels were rising. RRT was discontinued once ammonia levels dropped below 150 mcg/dL.

### 2.2. Standard Medical Treatment

Standard medical treatment in the ICU included elevating the head end of the bed to 30°, maintaining fluid balance with a target mean arterial pressure of 65–70 mmHg, and initiating mechanical ventilation if the Glasgow Coma Scale (GCS) dropped below 8. The target serum sodium range was 140–150 meq/L, and 3% hypertonic saline infusion was administered as needed. Mannitol was used when clinically indicated. Neurological assessments were conducted by a neurologist every 12 h or in case of any change in neurological status. Antiepileptic medications were not routinely administered and were given only if seizures were clinically evident. In the no-RRT group, CT scans of the brain were performed selectively to minimize patient movement, while in the RRT group, at least one CT scan was done during transfer to the SICU. Repeat scans were done in intubated patients if there was a change in neurological status. Intracranial catheter placement and therapeutic hypothermia were not part of the standard practice. HE was diagnosed according to the West Haven criteria [16].

### 2.3. Transplant Operation

The LT operation followed a standard protocol, including high hilar dissection, temporary portocaval shunt, and explant hepatectomy using the piggyback technique. Contraindications to LT include the absence of brain stem reflexes, brainstem herniation on imaging, the need for multiple inotropes, severe cardiopulmonary comorbidities, and pan-drug resistant sepsis.

### 2.4. Statistical Analysis

For this study, patients were divided into two groups: the no-RRT group (transplanted between April 2012 and February 2021) and the RRT group (transplanted between March 2021 and March 2023). The study outcome was 1-year posttransplant survival after LDLT. Categorical data were presented as numbers and percentages, and chi-square and Fisher's exact tests were used for significance. Continuous data were presented as median with interquartile range (IQR), and the Mann–Whitney *U* test was used for comparison. The Kaplan–Meier curves were used to report 1-year overall survival (OS), and the log-rank test was used to determine significance. A *p* value < 0.05 was considered statistically significant, and all analyses were performed using the Statistical Package for the Social Sciences (SPSS V.22 IBM).

## 3. Results

### 3.1. Patient Characteristics

**Figure 2:**
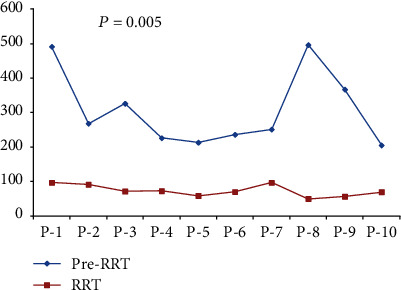
The serum ammonia level (micrograms per deciliter) in 10 patients in the renal replacement therapy group before and after renal replacement therapy.

Out of 24 transplants, seven (29.1%) were performed for patients in the pediatric age group. Hepatitis A was the etiology in 8/24 (33.3%) patients, and 17/24 (70.8%) patients required intubation with mechanical ventilation due to low GCS (Table 1). The median time to transplant was 3 days (2–4.5) in the no-RRT group and 2 days (2–3) in the RRT group (*p* = 0.361). Pretransplant inotropic support was required in 0/13 (0%) patients in the no-RRT group and 7/11 (63.6%) patients in the RRT group (*p* = 0.001). The median serum ammonia level at the time of transplant was 259.5 mcg/dL (222.7–398) in the no-RRT group and 70.6 mcg/dL (58.1–92.6) in the RRT group (*p* = 0.005). In the RRT group, serum ammonia levels < 100 mcg/dL were achieved in 10/10 (100%) patients before LT (Figure 2). One patient in this group did not meet the criteria for RRT.

### 3.2. Donor Characteristics

In nine out of 24 (37.5%) transplants, more than one donor workup was performed. Reasons for donor rejection included fatty liver (*n* = 3), periportal edema on CT (*n* = 1), refusal to donate after workup (*n* = 2), Hepatitis B surface antigen positivity (*n* = 1), pulmonary changes due to heavy smoking (*n* = 1), and changes in electrocardiogram (*n* = 1). Right lobe grafts were used in 21 out of 24 (87.5%) patients. Significant differences were observed in cold ischemia time, warm ischemia time, and duration of surgery between the groups (*p* < 0.05). Reconstruction of more than two outflow veins was done in two out of 11 (18.2%) patients in the no-RRT group and nine out of 10 (90%) patients in the RRT group who received right lobe grafts (*p* = 0.004). Among the 13 patients in the no-RRT group, one (7.7%) required intraoperative RRT due to renal insufficiency, while eight out of 11 (72.7%) patients in the RRT group needed intraoperative RRT (*p* = 0.002).

### 3.3. LT Outcomes

**Figure 3:**
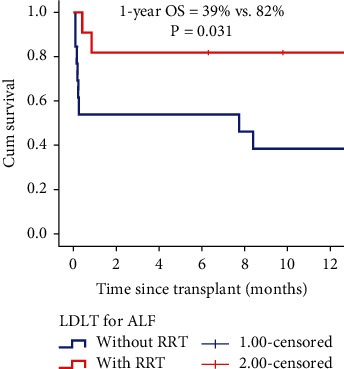
Posttransplant 1-year survival in patients with acute liver failure in the no-RRT (2012–2021) and RRT (2021–2023) groups.

After the transplant, seven out of 13 patients (53.8%) in the no-RRT group and all 11 patients (100%) in the RRT group were successfully extubated with full consciousness restored (*p* = 0.013) (Table 2). The 90-day mortality rates were 46.1% (six out of 13) in the no-RRT group and 18.1% (two out of 11) in the RRT group (*p* = 0.211). Brainstem herniation was the cause of death in 83.3% (five out of six) of no-RRT patients and 0% (zero out of two) of RRT patients (*p* = 0.03). In the RRT group, the two deaths were due to graft dysfunction and myocardial infarction (MI). One patient underwent retransplantation for graft dysfunction but died on the 26th postoperative day. The second patient with a recent history of MI developed ALF and required urgent LT. Despite an uneventful postoperative course, the patient experienced a repeat MI on the 13th postoperative day, leading to in-hospital death. The 1-year OS was 39% in the pre-RRT group and 82% in the RRT group (*p* = 0.031) (Figure 3). There were no major donor-related complications or mortality.

### 3.4. ALF Outcomes in the RRT Era

**Figure 4:**
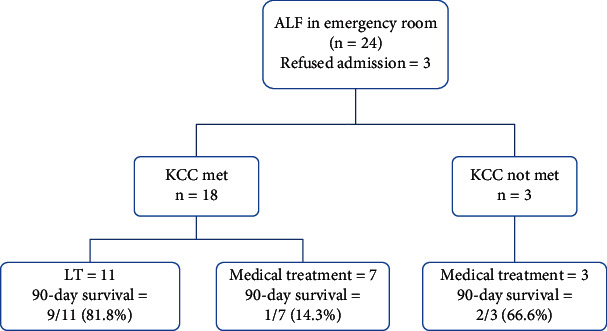
Outcomes in all patients admitted with acute liver failure between 2021 and 2023.

Between March 2021 and 2023, 21 patients with ALF were admitted from the emergency department (Figure 4). Out of these patients, 18 met the KCC and were offered LDLT. Eleven patients underwent LDLT, while seven patients did not undergo transplants due to family preference or lack of available donors. Among these seven patients, two required RRT, and only one of them (14.3%) survived. Among the three patients who did not meet the KCC, two (66.7%) had spontaneous recovery.

## 4. Discussion

The study demonstrates that RRT in the peritransplant period can improve posttransplant outcomes by reducing the risk of cerebral complications. A streamlined ALF pathway, including screening in the emergency room, prompt completion of the transplant workup, and timely LT, can expedite the evaluation process and lead to better outcomes. Patients meeting the KCC criteria have a low chance of survival without LT, and RRT should be used as a bridge to LT in these cases.

The 1-year OS in the no-RRT group was lower compared to recent studies on LDLT for ALF [17–19]. This was likely due to late presentation, cerebral edema, the need for mechanical ventilation, and relatively high MELD scores. The median serum ammonia level in the no-RRT group was 259.5 mcg/dL versus 70.6 mcg/dL in the RRT group (*p* = 0.005). In the no-RRT group, 5/6 (83.3%) early deaths were attributed to brainstem herniation. Cerebral edema, Grades 3 and 4 encephalopathy, and high MELD score are well-known risk factors for posttransplant death in ALF [17–19]. We believe that prolonged illness and poor control of hyperammonemia contributed to poor outcomes in the no-RRT group. There were no changes in our transplant selection criteria for ALF over the study period. In fact, more complex patients underwent LT in the later part of the study, with 9/11 (81.8%) requiring mechanical ventilation and 7/11 (63.6%) needing inotropic support at the time of transplant, as shown in Table 1.

RRT has been shown to improve 21-day transplant-free survival in patients with ALF [11, 12]. Hyperammonemia is a well-established risk factor for cerebral edema and Grades 3 and 4 HE. Serum ammonia levels of 150–200 mcg/dL are associated with inferior outcomes [9, 20]. Standard medical treatment has limited benefits in controlling hyperammonemia, and the recent American College of Gastroenterology guidelines recommend early initiation of RRT in ALF [21]. However, the impact of this strategy on posttransplant outcomes is unclear. Previously, studies have reported poor posttransplant outcomes in patients who underwent RRT for renal failure compared to those who underwent LDLT without RRT. These studies included a small number of patients, and hyperammonemia was not the focus of these studies [22, 23]. Our study is aimed at assessing the impact of correcting hyperammonemia on brainstem-related and overall posttransplant mortality in ALF patients. The median serum ammonia level in the no-RRT group was 259.5 mcg/dL, which could increase the risk of brainstem herniation during transplantation. Changes in cerebral perfusion during transplantation can lead to a rise in intracranial pressure during the preclamping phase and reperfusion stages of the recipient operation [24, 25]. Therefore, better control of ammonia levels before transplantation is essential to reduce the risk of intraoperative brain damage.

Various treatment options, such as plasma exchange, albumin dialysis, and RRT, have been used in patients with ALF, but their effectiveness is uncertain [1, 2]. In ALF, factors like systemic inflammatory response syndrome, infections, inotropic support, cerebral edema, and a high MELD score contribute to posttransplant mortality. Brainstem herniation was the main cause of posttransplant death in patients who did not receive RRT before transplantation. Early initiation of RRT in patients with high serum ammonia levels (≥200 mcg/dL) helped prevent brainstem herniation and improved posttransplant outcomes significantly. Intermittent hemodialysis can worsen cerebral edema due to changes in blood pressure, while RRT causes minimal blood pressure fluctuations and is well-tolerated by patients on low-dose inotropic support.

In patients with ALF who met the KCC but did not undergo LT, the outcomes were poor, with a high mortality rate. Patients who were eligible for LDLT but could not undergo the procedure eventually died (Figure 4). The challenges of arranging a living donor in a short period, especially in centers that exclusively perform LDLT, can be a major obstacle [26]. In some cases, patients may receive a borderline donor liver with suboptimal quality to save their lives. The study also highlights the unique challenges of voluntary liver donation in the context of ALF, including coercion and donor reluctance [26–28]. One of the donors in our cohort refused to donate after the workup was complete and felt he was not ready for surgery.

The study's main limitation is its retrospective design. However, the findings are significant as they focus on LDLT and the use of RRT in ALF patients. Delays in accessing RRT machines in large quaternary care hospitals with critical patients from various specialties could impact posttransplant outcomes, but this aspect was not evaluated in the study. Despite these limitations, the study demonstrates the benefits of LDLT and the positive effects of RRT in this patient population.

## 5. Conclusions

The study demonstrates that using RRT decreases perioperative serum ammonia levels and may lower posttransplant mortality caused by brainstem herniation. As our understanding of the mechanisms in ALF grows, better patient selection and RRT-supported transplantation could further improve outcomes.

## Figures and Tables

**Table 1:** Pretransplant and operative variables in patients who underwent living donor liver transplantation for acute liver failure.

**Variables**	**No-RRT group (** **n** = 13**)**	**RRT group (** **n** = 11**)**	**p** ** value**
Age, median (IQR) (years)	21 (15–27)	33 (13–46)	0.361
Etiology, *n* (%)			0.112
Hepatitis A	4 (30.8)	4 (36.3)	
Unknown	4 (30.7)	1 (9.1)	
Drug-induced	1 (7.7)	3 (27.3)	
Hepatitis E	1 (7.7)	2 (18.2)	
Budd–Chiari syndrome	1 (7.7)	—	
Wilson's disease	1 (7.7)	—	
Autoimmune	1 (7.7)	1 (9.1)	
MELD score, median (IQR)	32 (30–40)	36 (29.2–39)	0.649
Mechanical ventilation, *n* (%)	8 (61.5)	9 (81.8)	0.386
Inotropic support, *n* (%)	0	7 (63.6)	0.001
Cerebral edema on CT, *n* (%)	2/9	2/11	1.000
Renal insufficiency, *n* (%)	1 (7.6)	3 (27.2)	0.3
Lowest GCS, median (IQR)	7 (3.5–11.5)	3 (3–7)	0.134
Platelet count, median (IQR) (*μ*L)	228,000 (90,000–320,000)	288,000 (159,000–383,000)	0.303
WBC count, median (IQR) (*μ*L)	10,900 (9700–16,400)	12,360 (8830–15,970)	0.733
AST on admission, median (IQR) (u/L)	407 (179–2368)	783 (453–2307)	0.186
ALT on admission, median (IQR) (u/L)	464 (295–1119)	1163 (362–2590)	0.072
Total bilirubin, median (IQR) (mg/dL)	24.7 (18–37.4)	13.2 (10–27)	0.106
Creatinine, median (IQR) (mg/dL)	0.69 (0.5–0.9)	0.8 (0.6–1.6)	0.424
Highest ammonia, median (IQR) (mol/L)	326 (214–410)	384 (252–426)	0.519
PT/INR	2.7 (2.4–6)	3.5 (2.6–4.73)	0.456
Time to transplant, median (IQR) (days)	3 (2–4.5)	2 (2–3)	0.361
Mannitol, *n* (%)	11 (84.6)	9 (81.8)	1.000
Sedatives, *n* (%)	10 (76.9)	7 (63.6)	0.659
Antiepileptics, *n* (%)	3 (23)	3 (27.2)	1.000
Hypertonic saline, *n* (%)	8 (61.5)	9 (81.8)	0.386
Number of donors evaluated			0.206
1	10 (76.9)	5 (45.4)	
2	3 (23.1)	6 (54.6)	
BMI, median (IQR) (kg/m^2^)	24.7 (23.3–28.7)	26.8 (22.2–30.1)	1.0
Graft type, *n* (%)			1.0
Right lobe graft	11 (84.6)	10 (90.9)	
Left lobe graft	1 (7.7)	0 (0)	
Left lateral sector graft	1 (7.7)	1 (9.1)	
Outflow veins in right lobe, *n* (%)			0.004
One	5 (45.4)	1 (10)	
Two	4 (36.4)	0	
Three	2 (18.2)	9 (90)	
GRWR, median (IQR)	1.05 (0.89–1.4)	1.1 (0.92–1.3)	0.865
Liver attenuation, median (IQR)	12 (8.85–20)	14 (1–14)	0.733
Cold ischemia time, median (IQR) (minutes)	45 (14–62)	64 (55–68)	0.030
Warm ischemia time, median (IQR) (minutes)	35 (30.5–53.5)	57 (43–71)	0.030
Blood loss, median (IQR) (mL)	1600 (1250–2400)	1200 (1000–3000)	0.820
Surgery duration, median (IQR) (minutes)	500 (480–570)	660 (660–780)	0.001
Reperfusion syndrome	3 (23)	3 (27.2)	1.000
Intraoperative RRT, *n* (%)	1 (7.7)	8 (72.7)	0.002

**Table 2:** Posttransplant outcomes in patients with acute liver failure.

**Outcomes**	**No-RRT group (** **n** = 13**)**	**RRT group (** **n** = 11**)**	**p** ** value**
ICU stay, median (IQR) (days)	10 (9–13)	9 (7–12)	0.910
Hospital stay, median (IQR) (days)	25 (25–31)	21 (17–25)	0.649
Posttransplant extubation, *n* (%)	7 (53.8)	11 (100)	0.013
Days to extubation, median (IQR)	3 (1–4)	3 (2–3)	1.000
Return to full GCS, *n* (%)	7 (53.8)	11 (100)	0.013
Major complications, *n* (%)	7 (53.8)	5 (45.5)	1.000
Brain failure	5 (38.4)	0	
Graft dysfunction	1 (7.7)	1 (9.1)	
Cardiac arrest	—	1 (9.1)	
Upper GI bleed	—	1 (9.1)	
Burst abdomen	1 (7.7)	1 (9.1)	
Subcapsular hematoma	—	1 (9.1)	
90-day mortality, *n* (%)	6 (46.1)	2 (18.2)	0.211
Brainstem herniation	5 (38.4)	0 (0)	
Graft dysfunction	1 (7.7)	1 (9.1)	
Cardiac arrest	0 (0)	1 (9.1)	
90-day brain death mortality, *n* (%)	5 (83.3)	0 (0)	0.030

## Data Availability

The data is available from the corresponding author upon reasonable request.

## References

[1] Ozturk N. B., Herdan E., Saner F. H., Gurakar A. (2023). A comprehensive review of the diagnosis and management of acute liver failure. *Journal of Clinical Medicine*.

[2] Stravitz R. T., Lee W. M. (2019). Acute liver failure. *Lancet*.

[3] Bernal W., Wendon J. (2013). Acute liver failure. *The New England Journal of Medicine*.

[4] McPhail M. J. W., Farne H., Senvar N., Wendon J. A., Bernal W. (2016). Ability of King’s College criteria and model for endstage liver disease scores to predict mortality of patients with acute liver failure: a meta-analysis. *Clinical Gastroenterology and Hepatology*.

[5] Kumar R., Anand U., Priyadarshi R. N. (2021). Liver transplantation in acute liver failure: dilemmas and challenges. *World Journal of Transplantation*.

[6] Flamm S. L., Wong F., Ahn J., Kamath P. S. (2022). AGA clinical practice update on the evaluation and management of acute kidney injury in patients with cirrhosis: expert review. *Clinical Gastroenterology and Hepatology*.

[7] Yang H. R., Thorat A., Jeng L. B. (2018). Living donor liver transplantation in acute liver failure patients with grade IV encephalopathy: is deep hepatic coma still an absolute contraindication? A successful single-center experience. *Annals of Transplantation*.

[8] Anand A. C., Nandi B., Acharya S. K. (2020). Indian National Association for the study of the liver consensus statement on acute liver failure (part 1): epidemiology, pathogenesis, presentation and prognosis. *Journal of Clinical and Experimental Hepatology*.

[9] Kok B., Karvellas C. J. (2017). Management of cerebral edema in acute liver failure. *Seminars in Respiratory and Critical Care Medicine*.

[10] Torres D. M., Stevens R. D., Gurakar A. (2010). Acute liver failure: a management challenge for the practicing gastroenterologist. *Gastroenterología y Hepatología*.

[11] Stravitz R. T., Fontana R. J., Karvellas C. (2023). Future directions in acute liver failure. *Hepatology*.

[12] Niranjan-Azadi A. M., Araz F., Patel Y. A. (2016). Ammonia level and mortality in acute liver failure: a single-center experience. *Annals of Transplantation*.

[13] Cardoso F. S., Gottfried M., Tujios S., Olson J. C., Karvellas C. J., US Acute Liver Failure Study Group (2018). Continuous renal replacement therapy is associated with reduced serum ammonia levels and mortality in acute liver failure. *Hepatology*.

[14] European Association for the Study of the Liver (2017). EASL clinical practical guidelines on the management of acute (fulminant) liver failure. *Journal of Hepatology*.

[15] Bhatti A. B. H., Qasim S. F., Zamrood Z. (2024). Patient selection for living donor liver transplantation in acute-on-chronic liver failure. *Journal of Clinical and Experimental Hepatology*.

[16] Vilstrup H., Amodio P., Bajaj J. (2014). Hepatic encephalopathy in chronic liver disease: 2014 practice guideline by the American Association for the Study of Liver Diseases and the European Association for the Study of the Liver. *Hepatology*.

[17] Pamecha V., Vagadiya A., Sinha P. K. (2019). Living donor liver transplantation for acute liver failure: donor safety and recipient outcome. *Liver Transplantation*.

[18] Mehrotra S., Mehta N., Rao P. S., Lalwani S., Mangla V., Nundy S. (2018). Live donor liver transplantation for acute liver failure: a single center experience. *Indian Journal of Gastroenterology*.

[19] Pamecha V., Patil N. S., Falari S. (2023). Live donor liver transplantation for pediatric acute liver failure: challenges and outcomes. *Hepatology International*.

[20] Clemmesen J. O., Larsen F. S., Kondrup J., Hansen B. A., Ott P. (1999). Cerebral herniation in patients with acute E liver failure is correlated with arterial ammonia concentration. *Hepatology*.

[21] Shingina A., Mukhtar N., Wakim-Fleming J. (2023). Acute liver failure guidelines. *The American Journal of Gastroenterology*.

[22] Ikegami T., Shirabe K., Soejima Y. (2012). The impact of renal replacement therapy before or after living donor liver transplantation. *Clinical Transplantation*.

[23] Robinson A. M., Karvellas C. J., Dionne J. C. (2020). Continuous renal replacement therapy and transplant-free survival in acute liver failure: protocol for a systematic review and meta-analysis. *Systematic Reviews*.

[24] Keays R., Potter D., O'Grady J., Peachey T., Alexander G., Williams R. (1991). Intracranial and cerebral perfusion pressure changes before, during and immediately after orthotopic liver transplantation for fulminant hepatic failure. *The Quarterly Journal of Medicine*.

[25] Jalan R., Olde Damink S. W., Deutz N. E. (2003). Moderate hypothermia prevents cerebral hyperemia and increase in intracranial pressure in patients undergoing liver transplantation for acute liver failure. *Transplantation*.

[26] Ogura Y., Kabacam G., Singhal A., Moon D. B. (2020). The role of living donor liver transplantation for acute liver failure. *International Journal of Surgery*.

[27] Eguchi S., Furukawa H., Uemoto S. (2016). Outcomes of living donor liver transplantation alone for patients on maintenance renal replacement therapy in Japan: results of a nationwide survey. *Transplantation direct*.

[28] Bhatti A. B. H., Naqvi W., Mohsan M. (2024). Long-term medical and quality of life outcomes among voluntary liver donors. *Journal of Gastrointestinal Surgery*.

